# Clinical Study on Acute Pancreatitis in Pregnancy in 26 Cases

**DOI:** 10.1155/2012/271925

**Published:** 2012-11-18

**Authors:** Cheng Qihui, Zhang Xiping, Ding Xianfeng

**Affiliations:** ^1^Department of Gynaecology and Obstetrics, Hangzhou First People's Hospital, Zhejiang, Hangzhou 310006, China; ^2^9th Ward, Breast Surgery, Zhejiang Cancer Hospital, Zhejiang, Hangzhou 310022, China; ^3^Department of General Surgery, Hangzhou First People's Hospital, Zhejiang, Hangzhou 310006, China; ^4^College of Life Sciences, Zhejiang Sci-Tech University, Xiasha No. 2 Road, Zhejiang, Hangzhou 310018, China

## Abstract

*Aim*. This paper investigated the pathogenesis and treatment strategies of acute pancreatitis (AP) in pregnancy. *Methods*. We analyzed retrospectively the characteristics, auxiliary diagnosis, treatment strategies, and clinical outcomes of 26 cases of patients with AP in pregnancy. *Results*. All patients were cured finally. (1) Nine cases of 22 mild acute pancreatitis (MAP) patients selected automatic termination of pregnancy because of the unsatisfied therapeutic efficacy or those patients' requirements. (2) Four cases of all patients were complicated with severe acute pancreatitis (SAP); 2 cases underwent uterine incision delivery while one of them also received cholecystectomy, debridement and drainage of pancreatic necrosis, and percutaneous jejunostomy. One case had a fetal death when complicated with SAP; she had to receive extraction of bile duct stones and drainage of abdominal cavity after induced abortion. The other one case with hyperlipidemic pancreatitis was given induced abortion and hemofiltration. *Conclusions*. The first choice of MAP in pregnancy is the conventional therapy. Apart from the conventional therapy, we need to terminate pregnancy as early as possible for patients with SAP. Removing biliary calculi and drainage is supposed to be considered for acute biliary pancreatitis. Lowering blood lipids treatment should be applied to hyperlipidemic pancreatitis or given to hemofiltration when necessary.

## 1. Introduction

Acute pancreatitis in pregnancy (APIP) is rare and occurs in approximately 1 in 1,000 to 3 in 10,000 births [1–3]. Characterized as acute onset, many complications and high mortality, it is difficult to make a diagnosis and practice treatment, therefore prone to have a misdiagnosis and delay treatment so that threaten the heath of mom-baby as a result of the onset during female special physiological period. It's very useful for the prediction to have an early diagnosis and treatment for APIP patients [2].

The commonest reasons of APIP are biliary disease and congenital or acquired hypertriglyceridemia, which can occur during any trimester but over a half occurs during the third trimester, and very rarely APIP is associated with preeclampsia-eclampsia or HELLP syndrome [4, 5]. Pregnancy-associated acute biliary pancreatitis is a challenging clinical entity in terms of diagnosis and management with risks to both the pregnancy and the developing fetus. Sun et al. reported that hyperlipidemic pancreatitis and biliary pancreatitis are the main causes of severe and mild disease, respectively. Severe acute pancreatitis (SAP) in pregnancy usually occurs in the third trimester, and the severely affected patients are more liable to develop a critical condition that results in a higher risk of intrauterine fetal death [6]. But McKay et al. found that there was no evidence of a specific link between pregnancy and pancreatitis, but there was a marked association between pancreatitis and gallstones [7]. Pregnancy-related hypertriglyceridemia is rare, but it can be life threatening in some patients with genetic susceptibility. Its pathophysiology is incompletely understood. Severity scoring and effective management remain challenging [8].

APIP often presents as an acute abdomen and can have a lethal effect on the mother and the fetus. It is important to be aware that APIP may be more severe, posing a survival threat even in the youngest patients. We present 26 cases of APIP who were admitted from March 1997 to December 2009 in our hospital, in order to investigate the pathogenesis and treatment strategies.

## 2. Material and Methods

### 2.1. Diagnostic Criteria

The diagnosis was based on a clinical diagnosis and grading criteria of AP established by the pancreatic group of the Surgical Society of the Chinese Medical Association for those cases before 2007 [9, 10] and a guideline (protocol) formulated by the pancreatic group of the Gastroenterology society of Chinese Medical Association for those cases after 2007 [11]. According to the above diagnostic criteria, among the 26 cases were 22 MAP and 4 SAP. The diagnosis of hyperlipidemic pancreatitis (HLP) was based on the following criteria: blood triglyceride level is higher than 11.3 mmol/L with clinical manifestation of AP, as well as there is between 5.56 and 11.30 mmol/L with chyle-like blood excluding any other etiology [12].

### 2.2. Clinical Information

Among the 26 APIP patients, there were an average age of 28.35 ± 5.00 (22–38) years, a mean length of hospital stay of 12.39 ± 10.00 days, and mean pregnancy weeks of 25.74 ± 17.00 (2 early, 10 middle, and 14 late). There were 3 cases with overeating fast food before APIP onset. The main clinical symptom was as follows: upper abdominal pain, nausea, and vomiting in 15 cases, ectopic pain of left lower back in 5 cases, and abdominal distension in 6 cases. The main outcome measures included clinical manifestation and auxiliary examination in the treated group and the control group. The etiology included biliary pancreatitis in 20 cases, hyperlipidemic pancreatitis in 3 cases, and, an unknown reason in 3 cases (mild cases). Results were expressed as Mean ± SD.

## 3. Examination, Treatment, and Outcomes

### 3.1. Auxiliary Examination

All patients had high blood and urine amylase levels, blood amylase 563.96 ± 582.60 U/L (normal value: 0–100 U/L) and urine amylase 7761.82 ± 3396.00 U/L (normal value: 0–500 U/L). Six cases had hyperglycemia with the maximum blood glucose contents of 26 mmol/L. The majority of the 26 cases had a leukocyte increase with a mean number of 15.32 ± 7.27 × 10^9^/L and a percentage of leukocyte with that of 85.22 ± 6.03%. B-ultrasound showed pancreas enlargement, decreased echo, and peripancreatic, pelvic, and abdominal cavity effusion in all patients. Among the 13 cases with biliary tract disease cholecystitis, cholecystolithiasis and bile duct stone were found in 4 cases while cholecystitis and cholecystolithiasis in 4 cases, cholecystolithiasis, and bile duct stone in 3 cases, choledocholithiasis in 3 cases, and congenital choledochal cyst in 1 case. CT scan displayed pancreas enlargement with partial necrosis, fuzzy boundary, disappearance of peripancreatic fat space, and ascites in 4 cases with SAP. The 4 cases with SAP, ERCP demonstrated obvious dilation of bile-pancreas duct and lower bile duct stones which were extracted in 1 case. Blood-gas analysis revealed respiratory alkalosis and metabolic acidosis in all SAP patients. The average content of triglyceride (normal value: 0.40–1.53 mmol/L), total cholesterol (normal value: 2.53–5.4 mmol/L), and blood amylase were 5.46 ± 6.14 mmol/L, 7.25 ± 4.47 mmol/L, and 563.96 ± 582.60 U/L for all APIP cases, respectively.

### 3.2. Treatment and Outcomes

#### 3.2.1. MAP in Pregnancy

We used the conventional therapy and enhanced fetal monitoring and miscarriage prevention with gastrointestinal decompression, ECG monitoring, inhibition of gastric acid secretion (pantoprazole and omeprazole), trypsin secretion (octreotide and somatostatin), and activity (aprotinin) on the basis of fasting, anti-inflammation (cephalosporin), fluid infusion, spasmolysis, and lowering blood lipid. Some scholars reported that octreotide and somatostatin can be effective in controlling some APIP patients and do not induce any malformation and do not affect foetal development, but do not have a large-scale clinical validation [13, 14]. We observed closely the change of uterine contraction and vaginal secretion with fetal movements counting, ECG monitoring, and B-ultrasound to prevent premature delivery. Intravenous magnesium sulfate was given to patients with threatened preterm labor to inhibit uterine contraction and maintain term pregnancy. Nine cases (8 cases are biliary pancreatitis while an unknown reason for 1 case) selected automatic termination of pregnancy because they were not satisfied with the therapeutic efficacy or worried about the drug side effect on the fetal development. Among the 9 cases, induced abortion was conducted in 5 cases (in 4 cases fetus died and in 1 case fetus survived) during 12–34 weeks while uterine incision deliveries in 4 cases (in 1 case fetus died and in 3 cases fetus survived) during 33–37 weeks. All patients were cured and discharged. 

#### 3.2.2. SAP in Pregnancy

We carried out conventional therapy including nutritional support, correction of water-electrolyte imbalance and acid-base disturbance, and protection of organ function as well as administration of insulin for hyperglycemia. Cases with late pregnancy were forced to be terminated without considering whether the fetus could survive. We would end pregnancy for those who had unsatisfied therapeutic efficacy. Biliary-pancreatic surgery should be given for those with biliary pancreatitis conditionally to eliminate the predisposing factor of bile reflux. 1 case of 35-week pregnancy diagnosed as biliary SAP with MOF symptoms including hyperthermia, dyspnea, tachycardia and oliguria and deep yellow and turbid ascites with the higher amylase of 8280 U/L as well as intrauterine fetal distress received uterine incision deliveries and cholecystectomy, debridement and drainage of pancreatic necrosis, and percutaneous jejunostomy. In this study, there was 1 case of 30^+^ weeks pregnancy diagnosed as biliary SAP complicated with acute pulmonary injury and diabetes mellitus deteriorated after the conventional therapy and then had uterine incision deliveries (neonatus died). Another case of 30-week with biliary pancreatitis took ERCP sphincterotomy and drainage of abdominal cavity after induced abortion because of a fetal death a week later. There was 1 case with hyperlipidemic pancreatitis (blood triglyceride level >26.54 mmol/L) at 25-week pregnancy which received induced abortion and hemofiltration because of threatened abortion (neonates died). All patients were cured and discharged.

## 4. Discussions

The relationship between AP and pregnancy is not quite clear. It's generally believed that APIP results from a synergy effect of several factors. The incidence of gestational pancreatitis in this series was one in 6,790 pregnancies [15]. APIP should be considered in the differential diagnosis of upper quadrant abdominal pain with or without nausea and vomiting [16, 17]. In this study, there are 2 cases with overeating as well as others without obvious predisposing factors. Currently, there are two principal mechanisms of APIP. One is biliary pancreatitis derived from the disease of cholecyst and biliary tract. According to the related statistics, the incidence of gallstone in pregnancy is between 2.5% and 4.2% [18]. Li et al. reviewed Sixth People's Hospital Affiliated to Shanghai Jiao Tong University (Shanghai, China) between 2005 and 2010; they found the major etiology of APIP was due to gallstone and cholecystitis [19]. Abdominal ultrasound and endoscopic ultrasound are ideal imaging techniques for diagnosing APIP because they have no radiation risk. Computed tomography, magnetic resonance cholangiopancreatography (MRCP), and endoscopic retrograde cholangiopancreatography (ERCP) should be used with caution. In the last decades the outcome of acute pancreatitis in pregnancy was much better, and perinatal mortality was less than 5% [3]. 

Another pathogenesis is hyperlipoidemia in pregnancy that can result in the microcirculation disturbance and hyperlipoproteinemia. As a consequence, an amount of fatty acid derived from degradation of blood triglyceride by high pancreatic lipase causes APIP by pancreatic ischemia and necrosis resulted from capillary thrombus and breakdown of vessel wall. The placental lactogen produced by syneytiotrophoblast in pregnancy can disassociate fat notably and release a sum of free fatty acids which cause acute adipose infiltration of acinar cells and fat embolism of pancreatic vessel that lead to pancreatitis and necrosis [20, 21]. In addition, SAP could be raised from Oddi's sphincterismus resulted from high level of mental stress in pregnancy [22, 23]. 

The fact that the augmented uterus compresses pancreatic and biliary duct is a key factor for the development of APIP. Terminating pregnancy is to eliminate the compression on one side and not taking the fetus into account; the fetus for treatment on the other side. Many great medications can be applied so that for enriching treatment options. The majority of cases with MAP in pregnancy can be cured by the conventional therapy with a good prognosis. But it remains controversial whether surgery is required or not. We think that the pancreatic capsule incision, partial pancreatectomy, and peripancreatic and peritoneal drainage can be used for cases with severe infection, necrosis, and peripancreatic, and peritoneal, effusion. If treated conservatively, pregnant patients with an biliary pancreatitis appear to have a high recurrence rate. Early surgical intervention is appropriate and safe and does not increase the length of the hospital stay. Since cholelithiasis is an important predisposing factor for APIP while biliary surgery has very few impacts on pregnant women and fetus with the development of medicine, some scholars suggest that women of late marriage should receive cholecystectomy before pregnancy as well as pregnant women at the mid trimester [24]. 

Endoscopy is a great breakthrough for biliary APIP to eliminate bile duct stone and reduce the reflux of pancreatic duct by sphincterotomy and ENBD placing with better therapeutic efficacy than the conventional therapy and decreased mortality [25]. The treatment of acute biliary pancreatitis during pregnancy remains controversial. ERCP is a safe procedure for pregnant women. It can be conducted for biliary stenting and subsequent clearance after deliveries [26]. The combination of MRCP, nonradiation ERCP, and immediate laparoscopic cholecystectomy can provide definite treatment and seems to put both mother and fetus at lower risk than what was presumed [27].

Data from our study indicates that MAP is not absolutely a sign for neither pregnancy termination nor cesarean section but aggravating SAP is. But in China, the national single child per couple policy has profound influence in each family. A couple can only have one child in their lifetime. The Chinese people must obey this rule; anyone violating it will face severe punishment; the most common punishment is fine. When APIP occurrs, the pregnant women and their other family members will pay much attention to the fetal development and would rather terminate pregnancy when the adverse factors may affect the fetus. In this group, induced abortion was conducted in 5 cases of the above nine MAP patients during 12–34 weeks while uterine incision deliveries were done in another 4 MAP cases during 33–37 weeks because of two main reasons: (1) pregnancy progressively aggravated the AP condition; (2) there was fear of some therapeutic drugs which can affect fetal development. This phenomenon is relatively common in China. We found many patients were treated with aprotinin, octreotide and sandostatin, spasmolytics, cephalosporin antibiotics, and some kind of traditional Chinese medicines. Among of them many drugs cannot get large-scale using in clinical validation for pregnant women, but the doctors have to use because of no other drugs can choose to cure those APIP patients. However, unless the fetus is too young or the labour goes well, we should consider saving lives of pregnant women at first and terminate pregnancy as soon as possible once the situation deteriorates or no relief occurrs

For APIP, most scholars advocate non-surgical treatment except for those following cases: (1) pancreatic abscess or infected effusion; (2) associated with other serious complications such as gastrointestinal perforation; (3) the situation deteriorates after active treatment of 2-3 d [27]. There were 3 cases with SAP grown better with the removal of the primary disease after the pregnancy termination in this study. We believe that observing urine and blood amylase dynamically is the key to the correct diagnosis. B-ultrasound can show pancreas enlargement, decreased echo, peripancreatic pelvic, and abdominal cavity effusion and detect cholecystolithiasis and bile duct stone. CT scan can indicate signs such as pancreatic necrosis and abscess as well as an amount of effusion, which is more valuable for determining the severity of the disease and necessary even if the fetus is exposed to X-ray. Goldberg and Hegele reviewed advances in the clinical management of pregnancy-related hypertriglyceridemia; they found some kinds of interventions; we think several methods can be used in APIP including (1) low-fat diet, (2) nutritional supplements, (3) oral prescription medications, and (4) therapeutic plasma exchange [28]. Altun et al. reported two cases of hypertriglyceridemia-induced AP during pregnancy, which were successfully treated by plasmapheresis [29]. We found one case with hyperlipidemic pancreatitis in this study was given hemofiltration; the patient was cured finally. We should observe the lipid level for pregnant women to prevent APIP caused by pregnancy-induced hyperlipidemia. For those with elevated lipid level, we ought to conduct a close followup and dietary adjustment to control hypertriglyceridemia or given to hemofiltration or plasmapheresis sometimes in order to prevent APIP.
